# APC/C^Cdh1^-Mediated Degradation of the F-Box Protein NIPA Is Regulated by Its Association with Skp1

**DOI:** 10.1371/journal.pone.0028998

**Published:** 2011-12-20

**Authors:** Christine von Klitzing, Richard Huss, Anna Lena Illert, Astrid Fröschl, Sabine Wötzel, Christian Peschel, Florian Bassermann, Justus Duyster

**Affiliations:** Department of Internal Medicine ΙΙΙ, Technical University of Munich, Munich, Germany; Université Paris-Diderot, France

## Abstract

NIPA (Nuclear Interaction Partner of Alk kinase) is an F-box like protein that targets nuclear Cyclin B1 for degradation. Integrity and therefore activity of the SCF^NIPA^ E3 ligase is regulated by cell-cycle-dependent phosphorylation of NIPA, restricting substrate ubiquitination to interphase. Here we show that phosphorylated NIPA is degraded in late mitosis in an APC/C^Cdh1^-dependent manner. Binding of the unphosphorylated form of NIPA to Skp1 interferes with binding to the APC/C-adaptor protein Cdh1 and therefore protects unphosphorylated NIPA from degradation in interphase. Our data thus define a novel mode of regulating APC/C-mediated ubiquitination.

## Introduction

Cell cycle transitions are regulated by the temporally controlled activity of kinase cascades and ubiquitin-mediated proteolysis of key regulatory proteins. Two types of E3 ligase complexes, the Cullin-RING E3 ligases, including SCF (Skp1/Cullin/F-box protein) complexes and anaphase promoting complex or cyclosome (APC/C), are essential for regulating cell cycle progression [1], [2].

Activation of the APC/C is dependent on mitosis-specific phosphorylation of several subunits [3]–[6] and on the sequential binding of the WD-repeat-containing proteins, Cdc20 and Cdh1 [7], [8], which are thought to recruit substrates to the core enzyme.

Targeting of substrates by the APC/C depends on short destruction motifs in their primary sequence. The most commonly found sequences are the destruction-box (D-box, RxxL) [9], [10] and the KEN-box (KENxxxN) [11]. However, recent reports have revealed several other motifs apart from these canonical sites [12]–[15].

NIPA (nuclear interaction partner of ALK) was originally identified by our group as a human nuclear protein in a screen for interaction partners of the activated anaplastic lymphoma kinase (ALK) receptor tyrosine kinase [16]. We subsequently characterized NIPA as an F-box like protein that defines a ubiquitin E3 ligase (SCF^NIPA^) which targets nuclear cyclin B1 for degradation and thereby contributes to the timing of mitotic entry. Intriguingly, phosphorylation of NIPA in late G2 phase leads to dissociation of NIPA from the SCF core complex, thus restricting activity of the SCF^NIPA^ complex to interphase [17], [18]. Here, we report that phosphorylated NIPA is degraded at mitotic exit in an APC/C^Cdh1^-dependent manner. This degradation is regulated by the cell-cycle-dependent binding of NIPA to the SCF core-protein Skp1 and represents a novel mode of regulating APC/C-mediated ubiquitination.

## Results

### The phosphorylated form of NIPA is degraded in late mitosis

**Figure 1:**
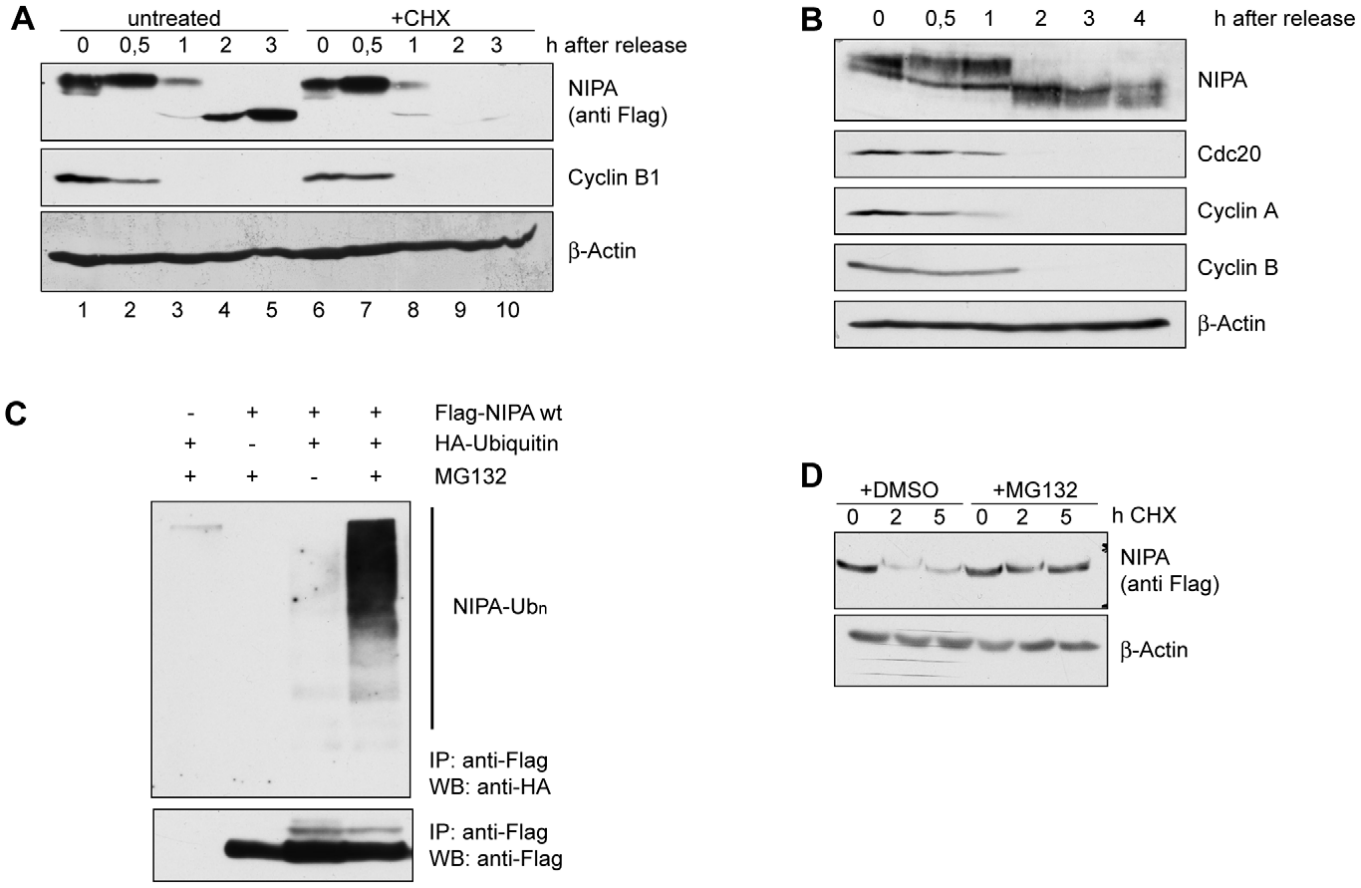
Phosphorylated NIPA is degraded during mitotic exit. (**A**) NIH3T3 cells stably overexpressing Flag-NIPA were arrested in prometaphase by a thymidine-nocodazole block and subsequently released into fresh medium either containing cycloheximide (CHX) or without supplements. Cells were collected at the indicated timepoints. The degree of synchronization was confirmed by analysis of the expression of cyclin B1 and FACS analysis (data not shown). (**B**) HeLa cells were arrested in prometaphase, released into fresh growth medium, and harvested for Western blot at the indicated timepoints. (**C**) HEK293T cells were transfected with (+) or without (-) HA-Ubiquitin and Flag-NIPA as indicated. Following treatment with MG132, extracts were prepared, denatured and subjected to Flag immunoprecipitation. Ub_n_: polyubiquitinated forms. (**D**) NIH3T3 cells retrovirally infected with a Flag-NIPA construct were treated with cycloheximide (CHX) and either DMSO or the proteasome inhibitor MG132. Cells were harvested for Western blot at the indicated timepoints.

Previous studies revealed phosphorylation of NIPA starting in late G2 phase of the cell cycle and peaking at the G2/M boundary (Fig. S1 and ref. [17]). After the G2/M transition, NIPA phosphorylation and expression levels decline precipitously upon entry into G1 and an unphosphorylated form of NIPA reappears later in G1 (Fig. 1A, lanes 1–5). Treatment of the cells with the translation inhibitor cycloheximide (CHX) after release from prometaphase prevented accumulation of the non-phosphorylated form of NIPA in G1 (Fig. 1A, lanes 6–10). This result indicates that the appearance of the lower form of NIPA is due to new protein synthesis rather than dephosphorylation of the upper form of NIPA and thus suggests that the phosphorylated form of NIPA is degraded in late mitosis. Remarkably, phosphorylated NIPA was degraded simultaneously with Cdc20, Cyclin B1 and Cyclin A, three known mitotic substrates of the APC/C (Fig. 1B).

To investigate whether NIPA degradation may be regulated by ubiquitination, we tested whether NIPA is polyubiquitinated *in vivo*. Therefore, cells were transfected with Flag-NIPA, HA-ubiquitin or with both. The transfected cells were treated with MG132 prior to harvesting. Immunoblotting detected high molecular weight ubiquitin conjugates in the Flag-NIPA immuno-complex in the presence of MG132 (Fig. 1C). To determine whether proteasomal function is required for NIPA degradation, NIPA-overexpressing cells were treated with cyclohexamide and the proteasome inhibitor MG132. Proteasome inhibition resulted in significant stabilization of NIPA (Fig. 1D). These results provide evidence that the ubiquitin-proteasome pathway controls the destruction of NIPA.

### NIPA is a substrate of APC/C^Cdh1^

**Figure 2:**
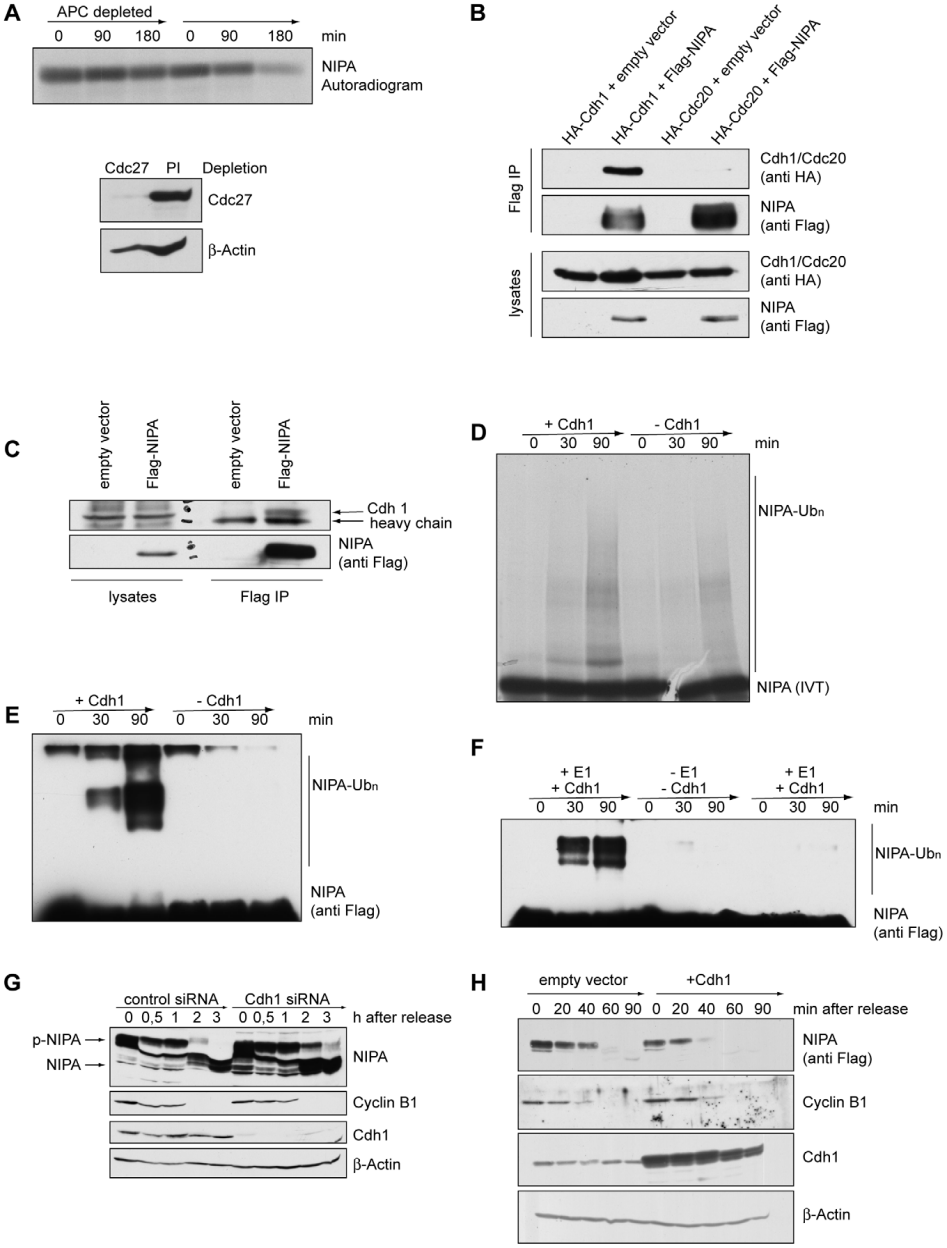
NIPA is degraded in an APC/C^Cdh1^-dependent manner. (**A**) Autoradiogram of ^35^S-labelled NIPA after incubation in HeLa cell extract prepared from G1 cells. Where indicated, the APC/C was depleted from the extract using a Cdc27 antibody. The Cdc27 Western blot shows efficient removal of Cdc27 from extracts. (**B**) Flag-NIPA and either HA-Cdh1 or HA-Cdc20 were expressed in HEK293T cells, and MG132 was added 6 h before the cells were collected. Cell extracts were immunoprecipitated (IP) with an antibody against Flag. (**C**) Flag-NIPA was expressed in HEK293T cells, and MG132 was added 6 h before the cells were collected. Cell extracts were immunoprecipitated (IP) with an antibody against Flag. (**D**) Autoradiogram of ^35^S-labelled NIPA after *in vitro* ubiquitination by APC/C immunoprecipitates derived from HeLa cells. Cdh1 was supplemented, where indicated. Ub_n_: polyubiquitinated forms. (**E**) and (**F**) Flag-NIPA was transiently expressed in HEK293T cells and immunoprecipitated using an agarose-bound anti-Flag antibody. Immunoprecipitates were used in APC *in vitro* ubiquitination reactions. Cdh1 and E1 ubiquitin-activating enzyme were supplemented as indicated. (**G**) HeLa cells were transfected with either control (firefly Luciferase) siRNA or NIPA siRNA, synchronized in prometaphase and subsequently released for the indicated times. (**H**) NIH3T3 cells stably overexpressing Flag-NIPA were transfected with empty vector or Cdh1, synchronized in prometaphase and then released for the indicated periods of time.

Since NIPA degradation occurs simultaneously with other APC/C-targets, we examined whether APC/C is required for NIPA degradation. To this end, we prepared extracts with high APC/C-activity and depleted APC/C from the extracts with antibody against Cdc27, a core subunit of APC/C, prior to *in vitro* degradation assays. We observed that exogenous, not Skp1-bound ^35^S-labeled NIPA was destroyed in the extract with APC/C activity, but was stabilized in the extract depleted by the Cdc27 antibody (Fig. 2A, upper panel). Western blot analysis confirmed that Cdc27 was successfully removed from the extract (Fig. 2A, lower panel).

The APC activator proteins Cdc20 and Cdh1 directly bind to their substrates to recruit them to the APC core complex [19]–[21]. To examine whether NIPA binds to one of these WD40 proteins *in vivo*, we performed co-immunoprecipitation studies. We found that NIPA binds to Cdh1 but not to Cdc20 (Fig. 2B, 2C and Fig. S2), suggesting that Cdh1 may mediate APC/C-dependent degradation of NIPA. Importantly, we were not able to show binding of NIPA and Cdh1 when only low expression of NIPA was observed (data not shown; see discussion).

To test whether NIPA was a substrate of APC/C, we examined whether immuno-purified APC/C directly catalyzed the ubiquitination of NIPA *in vitro*. NIPA was ubiquitinated by the APC/C *in vitro* and this ubiquitination was promoted by the addition of Cdh1 (Fig. 2D and 2E). The absence of the high-molecular weight forms of NIPA in samples in which E1 ubiquitin-activating enzyme was omitted from the reaction confirms that they present ubiquitin conjugates of NIPA (Fig. 2F).

To examine whether APC/C^Cdh1^ regulates NIPA stability *in vivo*, we studied the kinetics of NIPA degradation after a decrease of APC/C activity through knockdown of Cdh1. Hela cells were transfected with small interfering RNA targeted to Cdh1 or a control siRNA and arrested at prometaphase. Mitotic cells were then released into fresh media and examined at various timepoints thereafter. We observed that knockdown of Cdh1 resulted in a significant stabilization of the phosphorylated form of endogenous NIPA, indicating that APC/C^Cdh1^ indeed promotes degradation of NIPA at the exit of mitosis (Fig. 2G).

To further substantiate the role of Cdh1 in regulating the degradation of NIPA *in vivo*, we overexpressed Cdh1 in cells stably expressing a Flag-NIPA construct. Following transfection, cells were synchronized by nocodazole treatment with a subsequent release from the mitotic block. We found that overexpression of Cdh1 clearly accelerated the degradation of NIPA during the mitotic exit phase (Fig. 2H). This suggests that Cdh1 acts as a rate-limiting factor for the degradation of phosphorylated NIPA.

Together, these observations suggest that APC/C^Cdh1^ mediates degradation of NIPA in mitotic exit.

### Identification of NIPA domains required for its degradation

**Figure 3:**
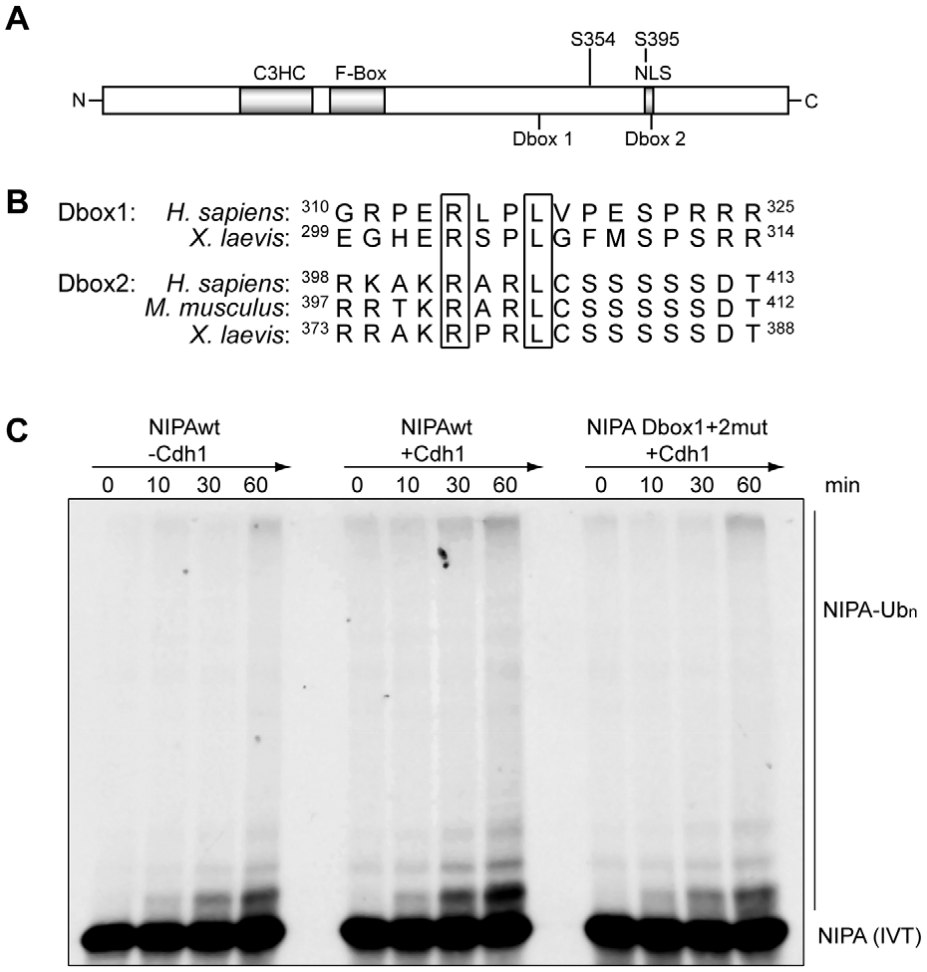
Degradation motifs in NIPA. (**A**) Schematic presentation of the NIPA protein, indicating the position of the D-box motifs. C: carboxyterminus; C3HC: Zinc-Finger motif; N: aminoterminus; NLS: nuclear localization signal; S: serine residue. (**B**) Alignment of NIPA D-box-like motifs in different species. C, Immunopurified APC/C supplemented with recombinant Cdh1 ubiquitinates wildtype NIPA *in vitro* but not the Dbox1+2 mutant.

Previous studies revealed that the APC/C recognizes particular destruction motifs in its substrates. By sequence analysis, we identified two putative D-box-like motifs in NIPA, whereof the second motif is conserved throughout human, mouse and *Xenopus laevis* (Fig. 3A,B). No other known putative Cdh1 recognition motifs were further identified. Mutation of these two D-box-like motifs leads to a decreased *in vitro* ubiquitination by the APC/C^Cdh1^ (Fig. 3C), indicating that these motifs are functional degradation motifs. However, the D-box mutant was not stabilized *in vivo* during mitotic exit (data not shown). The second D-box motif partially overlaps with the nuclear localization signal of NIPA and mutation of this putative degradation motif leads to cytoplasmic relocalization of the NIPA protein (Fig. S3), likely interfering with proper ubiquitination of NIPA.

*In vitro* binding assays identified amino acids 395–402 of NIPA as the relevant Cdh1-binding site (Fig. S4). This region also harbors the nuclear localization signal and the substrate binding site for Cyclin B1 [16], [18].

### Binding to Skp1 protects NIPA from APC/C^Cdh1^-mediated ubiquitination

**Figure 4:**
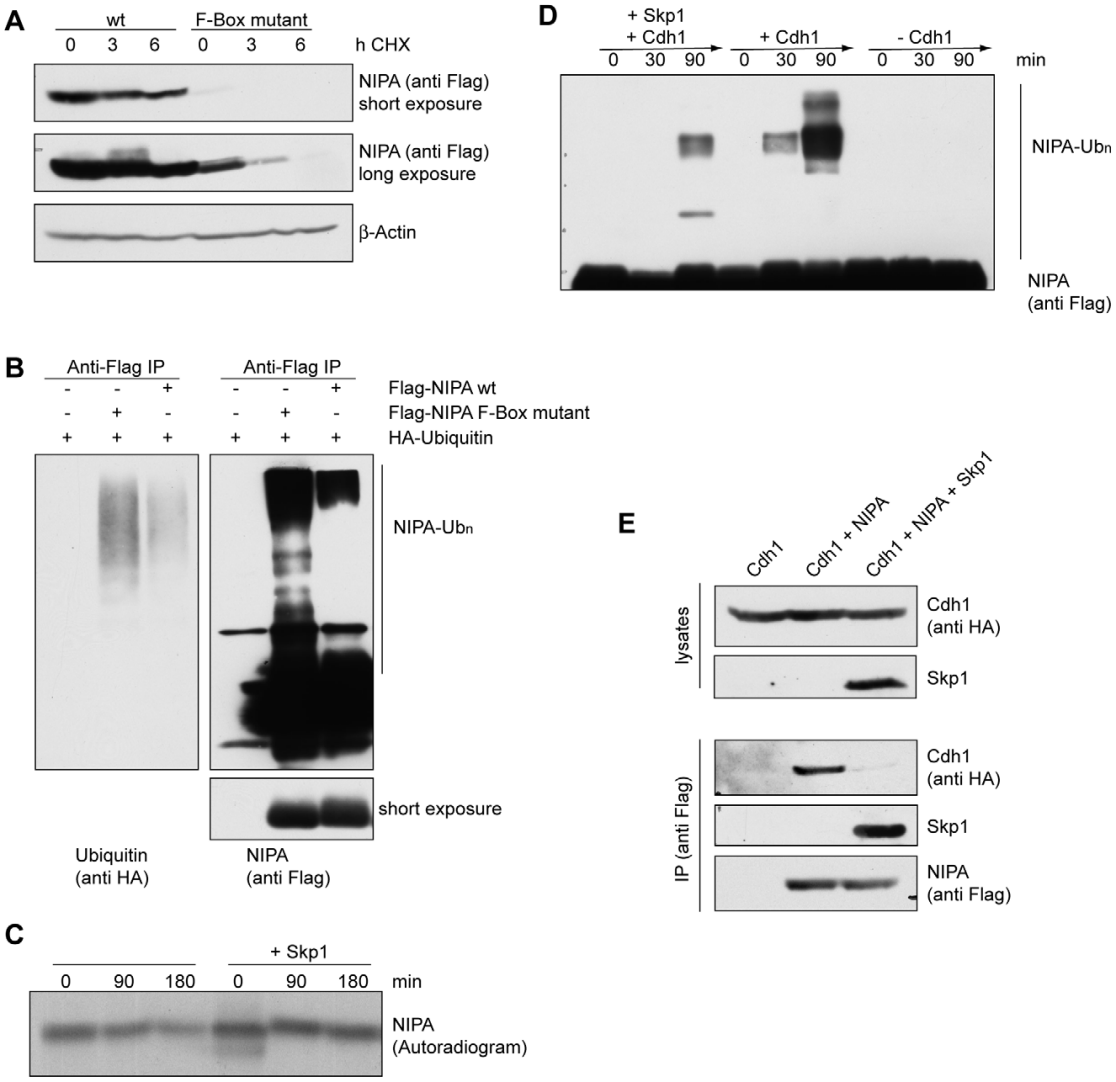
Binding to Skp1 protects NIPA from APC/C^Cdh1^-mediated degradation. (**A**) HEK293T cells transfected with Flag-NIPA wt or the F-Box mutant were treated with cycloheximide (CHX) for the indicated times and analyzed for total NIPA levels by immunoblotting. (**B**) HEK293T cells were transfected with HA-Ubiquitin and different amounts of Flag-NIPA wt or F-Box mutant to balance their expression. Following treatment with MG132, extracts were prepared, denatured and subjected to Flag immunoprecipitation. (**C**) Autoradiogram of an *in vitro* degradation assay using HeLa cell extract prepared from G1 cells. ^35^S-labelled Flag-NIPA was incubated with GST-Skp1 or GST and subsequently immunoprecipitated with agarose-bound anti-Flag antibody before being used in degradation assays. (**D**) Flag-NIPA was transiently expressed in HEK293T cells and immunoprecipitated using an agarose-bound anti-Flag antibody. Immunoprecipitates were used in APC *in vitro* ubiquitination reactions. Cdh1 and Skp1 were supplemented, where indicated. (**E**) HA-Cdh1 was co-expressed with Flag-NIPA and Skp1 in HEK293T cells as indicated. Cell extracts were immunoprecipitated (IP) with an antibody against Flag and analysed by immunoblotting.

The APC/C^Cdh1^ complex is active from late anaphase until late in G1. As unphosphorylated NIPA accumulates during this period of the cell cycle, a mechanism should exist, which protects the unphosphorylated form of NIPA from degradation, while phosphorylated NIPA is readily targeted by the APC/C^Cdh1^ complex. Since the phosphorylation status of NIPA regulates its binding to the SCF core protein, we hypothesized that the binding to Skp1 might control stability of NIPA. In accordance with this hypothesis, a mutant of NIPA, which is impaired in its binding to Skp1 by a mutation in the F-Box motif [17], has a significantly reduced stability compared to the wildtype protein (Fig. 4A). This reduced stability is associated with a more efficient ubiquitination of this mutant *in vivo* (Fig. 4B).

We therefore next tested whether binding to Skp1 protects NIPA from degradation *in vitro*. To this end, *in vitro* translated Flag-NIPA was incubated with purified GST-Skp1 to allow binding prior to *in vitro* degradation assays. As shown in Figure 4C, pre-incubation with GST-Skp1 protects NIPA from degradation in G1-synchronized cell extracts. Furthermore, addition of purified Skp1 to *in vitro* ubiquitylation reactions greatly reduced APC/C^Cdh1^-mediated in vitro ubiquitylation of NIPA (Fig. 4D). These results suggest that binding to Skp1, rather than phosphorylation of NIPA itself, regulates degradation of NIPA by the APC/C^Cdh1^ complex.

To further define the mechanism by which Skp1 regulates NIPA degradation, we investigated whether Skp1 and Cdh1 compete for binding with NIPA. To this end, we performed co-immunoprecipitation assays of Flag-NIPA and HA-Cdh1 in cells overexpressing Skp1 or not. As shown in Figure 4E, co-expression of Skp1 inhibits binding of NIPA and Cdh1.

We thus conclude that binding of NIPA to Skp1 protects it from APC/C^Cdh1^-mediated degradation by interfering with the interaction of NIPA with Cdh1. Phosphorylation of NIPA, however, leads to dissociation of NIPA from Skp1 and therefore allows recognition of NIPA by the APC/C^Cdh1^-complex and consequently degradation of NIPA. This provides a mechanistic explanation of how unphosphorylated NIPA is able to accumulate during G1 phase of the cell cycle, while phosphorylated NIPA is targeted by the APC/C^Cdh1^-complex.

## Discussion

At the G_2_/M transition, the NIPA F-box protein is phosphorylated on several serine residues [18]. This phosphorylation leads to dissociation of the SCF^NIPA^ E3 ligase. In late mitosis, the phosphorylated form of NIPA disappears, while simultaneously a nonphosphorylated form of NIPA emerges.

Here we show that the phosphorylated form of NIPA is degraded by the ubiquitin-proteasome system in late mitosis. It has been shown before that certain F-Box proteins are themselves ubiquitinated and degraded. Initially, this was attributed to an auto-ubiquitination reaction of the F-Box proteins [22]. However, for the two F-Box proteins Skp2 and Tome-1 an SCF-independent, but rather APC/C^Cdh1^-dependent degradation was shown recently [23]–[25]. Here, we further establish NIPA as a target of the APC/C^Cdh1^ *in vitro* and *in vivo*.

The APC/C^Cdh1^ is active during the G_1_ phase. However, unphosphorylated NIPA accumulates during this phase of the cell cycle, indicating that the APC/C^Cdh1^ exclusively ubiquitinates the phosphorylated form of NIPA, while the unphosphorylated form is protected from recognition by the APC/C^Cdh1^.

This regulation of an APC/C substrate by phosphorylation is remarkable since it was assumed until recently that APC/C-mediated ubiquitination is regulated by the activity of the ligase itself. Nevertheless, several reports recently showed a regulation of APC/C-mediated ubiquitination by substrate modification (for example see refs. 25–27). For NIPA we show however, that not phosphorylation itself, but the phosphorylation-induced dissociation from the SCF core protein Skp1 targets NIPA for degradation. In line with this model, mutation of the F-Box like motif in NIPA, which abolishes its binding to Skp1, greatly reduces the stability of the NIPA protein.

Wei *et al.* reported that a Skp2 F-box mutant that cannot form SCF complexes is a better APC/C^Cdh1^ substrate than wild-type Skp2 *in vivo* [23]. Strikingly, APC/C^Cdh1^-mediated ubiquitination of Skp2, similarly to NIPA, is regulated by timely phosphorylation of Skp2 [26]. However, it seems that this phosphorylation of Skp2 does not influence its binding to the SCF core complex [27], [28], therefore the cell-cycle dependent ubiquitination of Skp2 by the APC/C does not appear to be regulated by its interaction with Skp1. Nevertheless, APC/C-mediated ubiquitination of Skp2 and other substrates might not be regulated by phosphorylation itself, but rather by phosphorylation-induced modifications of interactions which regulate APC/C-dependent ubiquitination as we have shown here for NIPA.

Despite exclusive degradation of the phosphorylated form of NIPA in late mitosis and G1, we were able to show APC/C^Cdh1^-dependent ubiquitination and degradation of the unphosphorylated form *in vitro*. This finding further supports the theory that phosphorylation of NIPA itself has no impact on its availability for the APC/C.

Similarly, we were able to observe ubiquitination of unphosphorylated NIPA and its interaction with Cdh1 *in vivo*. However, this was only possible if large amounts of NIPA were overexpressed, indicating that ubiquitination of unphosphorylated NIPA can only take place if NIPA is present in excess compared to the levels of Skp1. This finding further supports our theory that Skp1 protects NIPA from degradation by interfering with recruitment of Cdh1.

We identified two putative destruction motifs in NIPA. Mutation of these motifs leads to a decreased Cdh1-dependent in vitro ubiquitination compared to the wildtype NIPA protein, indicating a role of these motifs for Cdh1-dependent ubiquitination. However, we were not able to show a stabilization of the mutant protein in vivo. This might be due to the fact that mutation of the second D-box motif leads to cytoplasmic relocalization of NIPA, likely interfering with proper ubiquitination of NIPA. Nonetheless, further as yet unidentified degradation motifs might be important for efficient degradation of NIPA in vivo.

In summary, our results provide evidence that the F-Box like protein NIPA is degraded in late mitosis in an APC/C^Cdh1^-dependent manner. We further show that binding to the SCF core complex protects NIPA from APC/C-mediated degradation, leading to exclusive degradation of the phosphorylated form of NIPA. We thus define a novel mode of controlling degradation of F-Box proteins, providing an additional layer of control over APC/C-mediated ubiquitination.

## Materials and Methods

### Plasmids, Antibodies and immunological procedures

Details of the construction of various NIPA plasmids are available from the authors upon request. Point mutations of the NIPA cDNA and deletion mutants were prepared using the Quickchange mutagenesis kit (Stratagene). HA-tagged Cdh1 and Cdc20 plasmids were kindly provided by M. Pagano and pcDNA3.1-Skp1 was provieded by Z–Q. Pan. pCMV-HA-Ubiquitin was a generous gift provided by W. Krek.

Anti-CDH1 antibody (Ab-2) was from Calbiochem and anti-Skp1 antibody was from Zymed. Anti-Flag and anti-β-Actin antibodies were from Sigma-Aldrich. Anti-CDC20 (H175), anti-CDC27 (AF3.1), anti-Cyclin A (H-432), anti-Cyclin B1 (H-433), anti-HA (Y-11) antibodies were from Santa Cruz. Anti-NIPA antibody was described before [17]. Immunoblot analysis and immunoprecipitations were performed as described [29].

### Cell Culture, Synchronization, Transfection and Treatment with Drugs

HeLa, NIH3T3 and HEK293T cells were cultivated in DMEM supplemented with 10% FCS and 2 mM L-glutamine. Transient transfections were performed using Lipofectamine 2000 (Invitrogen) transfection reagents. Stable NIH 3T3 cell lines have been described before [17]. Cells were synchronized in prometaphase by sequential culture with 2 mM thymidine for 12 h and 500 ng/ml nocodazole for 10–12 h.

To inhibit protein synthesis, cells were cultured in the presence of 50 µg/ml cycloheximide and to inhibit proteasomal degradation, cells were cultured in the presence of 10 µM MG132.

### siRNA

siRNAs were purchased from Proligo and transfected into subconfluent HeLa cells using Lipofectamine 2000 (Invitrogen) according to the manufacturer's instructions. The target sequence of Cdh1 siRNA was 5′-AATGAGAAGTCTCCCAGTCAG-3′ [30]. A firefly luciferase siRNA served as control.

### GST-fusion proteins and pull-down assays

Skp1, NIPA wt and all NIPA deletion mutants were expressed in E.coli (BL-21) using pGEX vectors (Amersham) and purified on Glutathion-S-Sepharose 4B beads (Amersham Pharmacia Biotech). If required, purified proteins were eluted with 20 mM glutathione in 100 mM Tris/HCl, pH 8.0, 120 mM NaCl.

For GST pull-down assays, 30 µl of *in vitro* translated, ^35^S-labeled Cdh1 was incubated with glutathione beads containing bound NIPA wt, NIPA deletion mutants or GST alone in binding buffer (10 mM Tris/HCl, pH 7.5, 100 mM NaCl, 5 mM EDTA, 0.1% Triton X-100, protease inhibitor cocktail) for 1 h at 4°C. Bound fractions were analyzed by SDS-PAGE prior to autoradiography.

### In vivo ubiquitination

For detection of *in vivo* ubiquitin-conjugates of ectopic NIPA, HEK293T cells were cotransfected with HA-tagged Ubiquitin and Flag-NIPA constructs. Cells were treated with either MG132 or DMSO 6 h before harvesting. Cell lysates were supplemented with denaturation buffer (1 mM DTT, 50 mM Tris-Cl (pH 7.5), 0.5 mM EDTA, 1% SDS final concentration) and denatured for 10 min at 95°C prior to immunoprecipitation with agarose-bound anti Flag antibody.

### In vitro ubiquitination assays

The APC/C was purified from exponentially growing HeLa cells as described before [31]. ^35^S-labeled NIPA was prepared by *in vitro* translation using a rabbit reticulocyte lysate (TNT, Promega). The labeled substrate was added to the *in vitro* ubiquitination reaction mix containing 40 mM Tris-Cl pH 7.6, 0.7 mM DTT, 5 mM MgCl_2_, 1 mg/ml ubiquitin, 10 µg/ml ubiquitin aldehyde, 0.84 µg/ml E1 ubiquitin-activating enzyme, 10 µg/ml UbcH10, 0.1 µg/ µl cycloheximide an ATP-generating system and APC/C-loaded beads. If indicated, baculovirus-produced Cdh1 was added to the reaction.

The reaction was incubated at 30°C and fractions were taken at indicated timepoints and diluted in SDS-sample buffer. The ubiquitinated forms of NIPA were analyzed by SDS-PAGE prior to autoradiography.

### In vitro degradation assays

Early G1 synchronized HeLa cells were harvested and the cell pellet was resuspended in lysis buffer (10 mM Tris-Cl, pH 7.5, 130 mM NaCl, 5 mM EDTA, 0,5%, Triton X-100, 20 mM Na_2_HPO_4_/NaH_2_PO_4_ (pH 7.5), 10 mM sodiumpyrophosphate (pH 7.0), 1 mM sodiumorthovanadate, 20 mM sodium-fluoride, 1 mM glycerol-2-phosphate and a protease inhibitor cocktail (Complete; Roche)).

For degradation assays, the clarified supernatant was supplemented with 1.5 mg/ml ubiquitin, 0.1 mg/ml cycloheximide, 20 mM DTT, 1 mM MgCl_2_ and an energy mix. 36 microliters of the extract was then added to four microliter of ^35^S-labeled substrate synthesized by *in vitro* translation (Promega). Reactions proceeded at 30°C for the indicated times, and the extent of degradation was determined by autoradiography.

In the immunodepletion assay, anti-Cdc27 or preimmuneserum and protein G beads were added to the extract for 2 h at 4°C. The beads were removed by centrifugation, and the supernatant was used for degradation assays. A portion of the extract was processed for Western blot analysis.

## Supporting Information

Figure S1 **NIPA is phosphorylated in mitosis.** Hela cells were synchronized in prometaphase by a sequential thymidine-nocodazole block. Cell extracts were either treated with acid potato phosphatase or left untreated and analyzed by immunoblotting using an anti-NIPA antibody.
(TIF)

Figure S2 **NIPA interacts with Cdh1.** Myc-NIPA and either HA-Cdh1 or HA-Cdc20 were expressed in HEK293T cells, and MG132 was added 6 h before the cells were collected. Cell extracts were immunoprecipitated (IP) with an antibody against HA-tag and analysed by immunoblotting.
(TIF)

Figure S3 **The Dbox-like motifs in NIPA.** **(A)** Overlap of the NLS and the Dbox2 motif in NIPA; amino acids 390–409 of the NIPA protein are shown. (**B)** Mutation of the Dbox2 motif interferes with correct nuclear localization of NIPA. Immunofluorescence of NIH/3T3 cells expressing Flag-tagged NIPA constructs.
(TIF)

Figure S4 **Mapping of the Cdh1-binding site in NIPA.** **(A)** schematic presentation of the NIPA deletion mutants assayed in (B). Binding of Cdh1 is indicated as +: binding similar to NIPAwt; -: no significant binding. C3HC: Zinc-finger motif; NLS: nuclear localization signal. **(B)** GST pulldown assays using various GST-NIPA deletion constructs and ^35^S-labelled, *in vitro* translated Cdh1.
(TIF)
