# Supplementary figures and images for: APC/CCdh1-Mediated Degradation of the F-Box Protein NIPA Is Regulated by Its Association with Skp1
Source: PLoS One. 2011 Dec 20;6(12):e28998. doi: 10.1371/journal.pone.0028998 (PMC3243670; doi:10.1371/journal.pone.0028998)

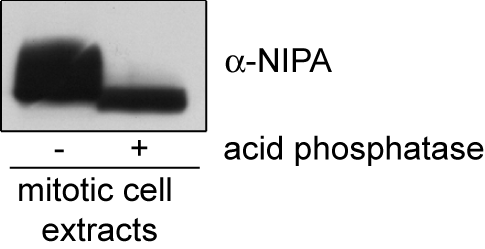

Supplement: Figure S1 — NIPA is phosphorylated in mitosis. Hela cells were synchronized in prometaphase by a sequential thymidine-nocodazole block. Cell extracts were either treated with acid potato phosphatase or left untreated and analyzed by immunoblotting using an anti-NIPA antibody. (TIF) [file pone.0028998.s001.tif]

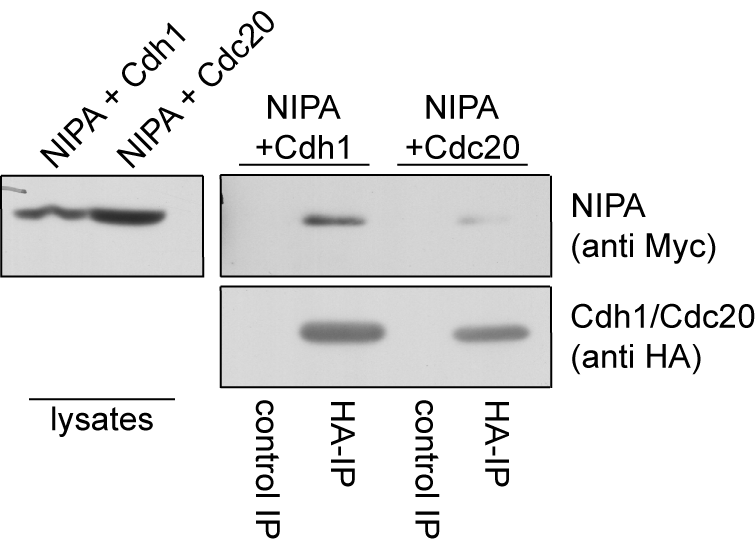

Supplement: Figure S2 — NIPA interacts with Cdh1. Myc-NIPA and either HA-Cdh1 or HA-Cdc20 were expressed in HEK293T cells, and MG132 was added 6 h before the cells were collected. Cell extracts were immunoprecipitated (IP) with an antibody against HA-tag and analysed by immunoblotting. (TIF) [file pone.0028998.s002.tif]

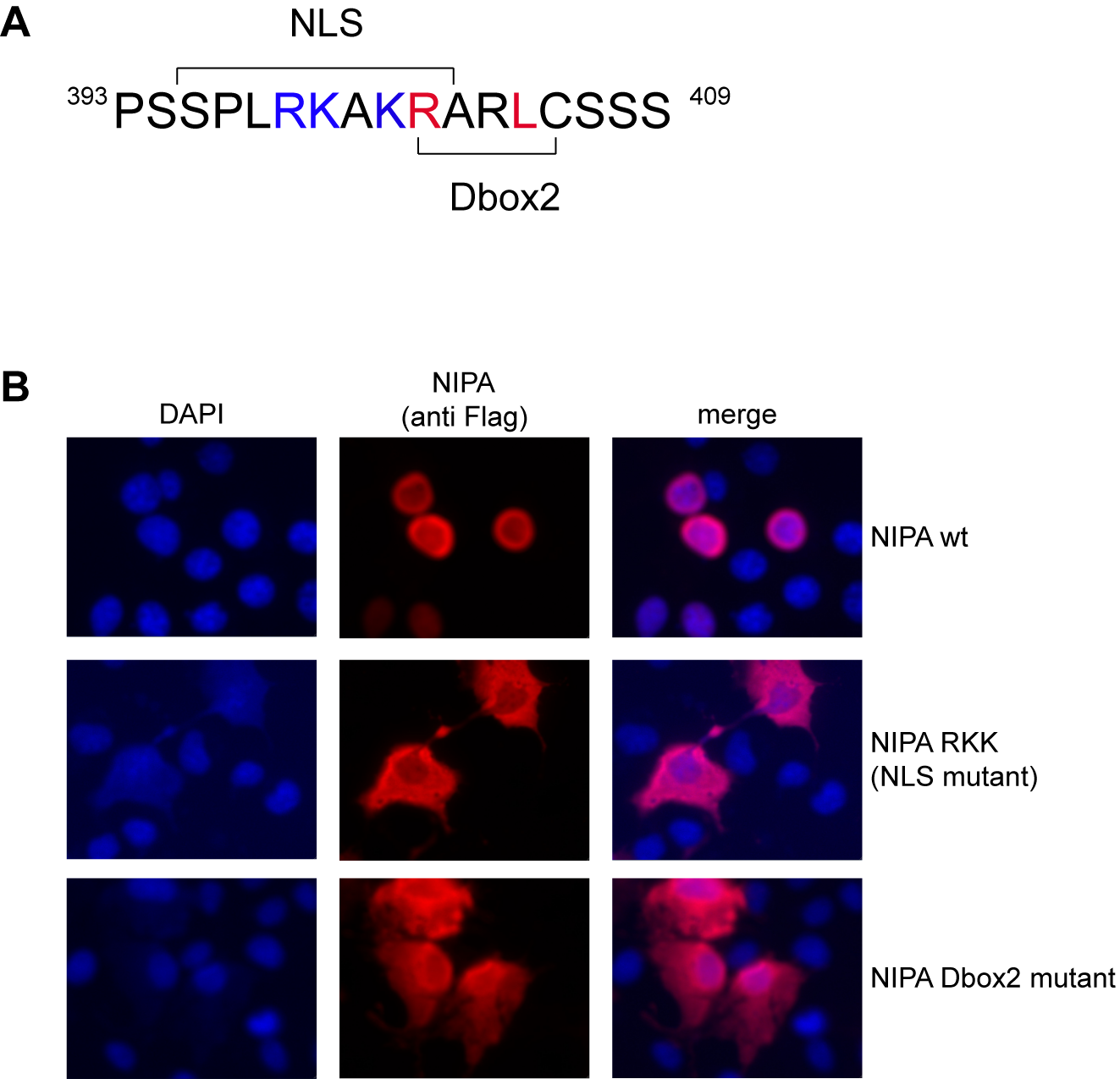

Supplement: Figure S3 — The Dbox-like motifs in NIPA. (A) Overlap of the NLS and the Dbox2 motif in NIPA; amino acids 390–409 of the NIPA protein are shown. (B) Mutation of the Dbox2 motif interferes with correct nuclear localization of NIPA. Immunofluorescence of NIH/3T3 cells expressing Flag-tagged NIPA constructs. (TIF) [file pone.0028998.s003.tif]

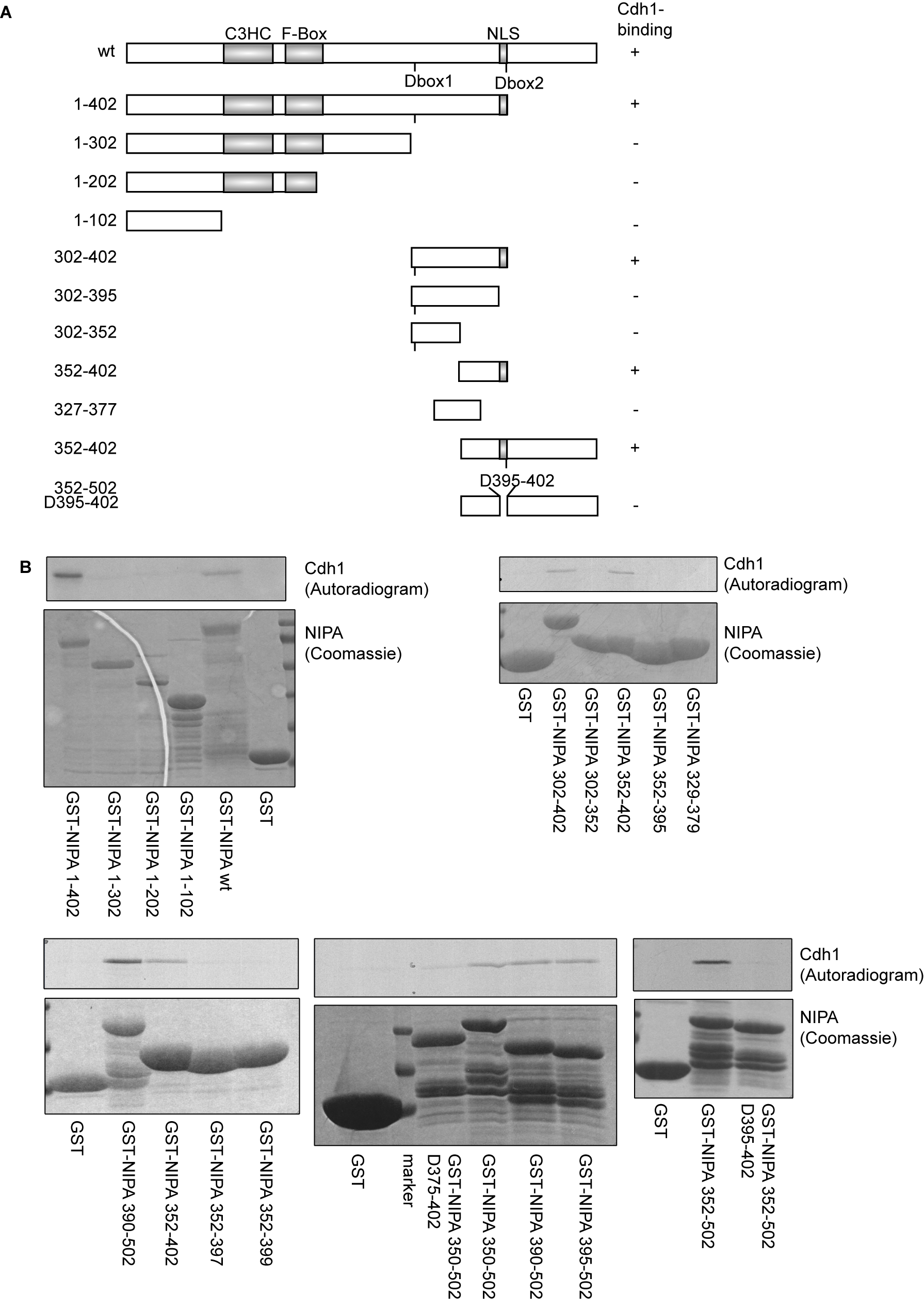

Supplement: Figure S4 — Mapping of the Cdh1-binding site in NIPA. (A) schematic presentation of the NIPA deletion mutants assayed in (B). Binding of Cdh1 is indicated as +: binding similar to NIPAwt; -: no significant binding. C3HC: Zinc-finger motif; NLS: nuclear localization signal. (B) GST pulldown assays using various GST-NIPA deletion constructs and 35S-labelled, in vitro translated Cdh1. (TIF) [file pone.0028998.s004.tif]
